# Post-COVID-19 syndrome: persistent symptoms, functional impact, quality of life, return to work, and indirect costs - a prospective case study 12 months after COVID-19 infection

**DOI:** 10.1590/0102-311XEN026623

**Published:** 2024-02-19

**Authors:** Fernando Shizuo Ida, Hebert Pereira Ferreira, Ana Karla Mendonça Vasconcelos, Iris Aline Brito Furtado, Cristina Janaina Pinheiro Morais Fontenele, Antonio Carlos Pereira

**Affiliations:** 1 Centro de Neurorreabilitação SARAH Fortaleza, Rede SARAH de Hospitais de Reabilitação, Fortaleza, Brasil.; 2 Faculdade de Odontologia de Piracicaba, Universidade Estadual de Campinas, Piracicaba, Brasil.

**Keywords:** COVID-19, Functional Status, Quality of Life, Costs and Cost Analysis, Absenteeism, COVID-19, Estado Funcional, Qualidade de Vida, Custos e Análise de Custo, Absenteísmo, COVID-19, Estado Funcional, Calidad de Vida, Costos y Análisis de Costo, Absentismo

## Abstract

The persistent symptoms of post-COVID-19 syndrome negatively impact health, quality of life, and productivity. This study aimed to describe the persistent symptoms of post-COVID-19 syndrome (especially neurological ones) and their 12-month post-infection cognitive, emotional, motor, quality of life, and indirect cost repercussions. Patients showing the first symptoms of COVID-19 from January to June 2021 who developed post-COVID-19 syndrome and sought care at the Fortaleza Unit (Ceará, Brazil) of the SARAH Network of Rehabilitation Hospitals were included in this study. Information was obtained at the baseline follow-up and by telephone interview 12 months post-infection. In total, 58 people participated in this study with an average age of 52.8±10.5 years, of which 60% required an ICU. The most frequent symptoms on admission included fatigue (64%), arthralgia (51%), and dyspnea (47%), whereas, after 12 months, fatigue (46%) and memory impairment (39%). The following scales/functional tests showed alterations: PCFS, MoCA, HAD, FSS, SF-36, TLS5x, timed up and go, 6-minute walk, and handgrip. Indirect costs totaled USD 227,821.00, with 11,653 days of absenteeism. Moreover, 32% of patients were unable to return to work. Better TLS5x and higher SF-36 scores in the functional capacity, physical functioning, vitality, and pain dimensions were associated with return to work (p ≤ 0.05). The most frequent persistent symptoms referred to fatigue, arthralgia, dyspnea, anxiety, and depression, which negatively affected cognitive, emotional, and motor function and quality of life. These symptoms lasted for over a year, especially fatigue and memory alteration, the latter of which being the most reported after COVID-19 infections. Results also show a significant difficulty returning to work and indirect costs of USD 4,847.25 per person/year.

## Abstract (pt)

Os sintomas persistentes da síndrome pós-COVID-19 acarretam impactos negativos na saúde, qualidade de vida e produtividade. O objetivo deste trabalho foi descrever os sintomas persistentes da síndrome pós-COVID-19, principalmente neurológicos, e as repercussões cognitivas, emocionais, motoras, de qualidade de vida e de custos indiretos, 12 meses após a infecção. Pacientes com primeiros sintomas entre janeiro e junho de 2021 evoluíram com síndrome pós-COVID-19 e procuraram atendimento na unidade da Rede SARAH de Hospitais de Reabilitação em Fortaleza, Ceará, Brasil. As informações foram obtidas no início do acompanhamento e por entrevista telefônica após 12 meses da infecção. Participaram do estudo 58 pessoas, com idade média de 52,8 anos (±10,5) e 60% permaneceram em UTI. Os sintomas mais frequentes na admissão foram: fadiga (64%), artralgia (51%) e dispneia (47%); e após 12 meses: fadiga (46%) e alteração de memória (39%). Foram identificadas alterações nas escalas/testes funcionais: PCFS, MoCA, HAD, FSS, SF-36, TLS5x, Timed up and go, caminhada de 6 minutos e preensão manual. Os custos indiretos totais foram de USD 227.821,00, com 11.653 dias de absenteísmo; 32% dos pacientes não voltaram a trabalhar. Melhores resultados de TLS5x e SF-36, nas dimensões capacidade funcional, aspecto físico, vitalidade e dor, demonstraram associação com retorno laboral (p ≤ 0,05). Os sintomas persistentes mais frequentes foram: fadiga, artralgia, dispneia, ansiedade e depressão, com repercussões negativas na funcionalidade cognitiva, emocional, motora e qualidade de vida. Esses sintomas perduraram por mais de um ano, principalmente fadiga e alteração de memória, sendo esta última mais relatada tardiamente. Houve dificuldade importante de retorno laboral e custos indiretos de USD 4.847,25 por pessoa/ano.

## Abstract (es)

Síntomas persistentes del síndrome post-COVID-19 provocan impactos negativos en la salud, calidad de vida y productividad. El objetivo fue describir los síntomas persistentes del síndrome post-COVID-19, sobre todo neurológicos, y las repercusiones cognitivas, emocionales, motoras, de calidad de vida y los costos indirectos, 12 meses tras la infección. Pacientes que tuvieron los primeros síntomas entre enero y junio de 2021, desarrollaron síndrome post-COVID-19 y buscaron atención en la unidad de la Red SARAH de Hospitales de Rehabilitación en Fortaleza, Ceará, Brasil. Las informaciones se obtuvieron al inicio del seguimiento y por entrevista telefónica 12 meses tras la infección. Participaron 58 personas, con edad de 52,8±10,5 años y el 60% permaneció en la UTI. Los síntomas más frecuentes al ingreso fueron: fatiga (64%), artralgia (51%) y disnea (47%); tras 12 meses fueron: fatiga (46%) y alteración de memoria (39%). Se identificaron alteraciones en las escalas/testes funcionales: PCFS, MoCA, HAD, FSS, SF-36, TLS5x, timed up and go, caminar por 6 minutos y prensión manual. Los costos indirectos totales fueron USD 227.821,00, con 11.653 días de absentismo. El 32% de los pacientes no volvió a trabajar. Mejor TLS5x y SF-36 en las dimensiones capacidad funcional, aspecto físico, vitalidad y dolor demostraron una asociación con el retorno al trabajo (p ≤ 0,05). Los síntomas persistentes más frecuentes fueron fatiga, artralgia, disnea, ansiedad y depresión, con repercusiones negativas en la funcionalidad cognitiva, emocional, motora y calidad de vida. Estos síntomas continuaron por más de un año, sobre todo la fatiga y la alteración de la memoria, siendo esta última reportada con más frecuencia tardíamente. Hubo una dificultad importante en el retorno al trabajo y costos indirectos de USD 4.847,25 persona/año.

## Introduction

Post-COVID-19 syndrome is defined as a symptom constellation during or after COVID-19 infections that persists for more than 12 weeks and are insufficiently explained by alternative diagnoses ^1^.

Studies have shown incidence rates of post-COVID-19 syndrome with different examination and follow-up times after acute infections. Tenforde et al. ^2^ estimated that more than 30% of individuals affected by COVID-19 developed post-COVID-19 syndrome (including asymptomatic cases), whereas Huang et al. ^3^ found the syndrome in 80% of hospitalized patients.

A wide range of persistent symptoms have been identified after mild and severe cases of COVID-19 infection ^4^, of which the most commonly reported include fatigue, dyspnea, anosmia, sleep disorders, arthralgia, headaches, cough, memory alterations, and impaired mental health ^3^ ^,^ ^4^ ^,^ ^5^ ^,^ ^6^ ^,^ ^7^.

In the United States, estimates suggest a USD 3,045 direct health care cost per COVID-19 case ^8^. However, indirect costs can represent a considerable proportion of the total economic cost of the disease and include the monetary value of productivity loss stemming from absence from paid work and other unpaid activities (e.g., caregiving) due to morbidity and mortality, with premature mortality being one of the main contributors to these costs ^9^.

This study aimed to describe persistent post-COVID-19 symptoms (especially neurological ones) and their repercussion on cognitive, emotional, and motor functions, quality of life, and indirect costs due to the loss of work productivity 12 months after acute infections.

## Materials and method

### Participants and study site

People with the first symptoms of COVID-19 from January to June 2021 who sought care at the Fortaleza Unit (Ceará, Brazil) of the SARAH Network of Rehabilitation Hospitals (SARAH Network) from April to June 2021 after the resolution of acute infections and who were diagnosed with post-COVID-19 syndrome were included in this study.

SARAH Network provides qualified and free medical care in neurology, orthopedics, and rehabilitation to all population strata. It has nine units in seven Brazilian states, including the Federal District.

During the COVID-19 pandemic, SARAH Network also aimed to rehabilitate patients with neurological complications due to COVID-19, such as post-COVID-19 syndrome, stroke, spinal cord inflammation, brachial plexus injuries, impaired muscle strength and/or sensation in upper or lower limbs, changes to balance and motor coordination, memory alterations, and other post-COVID-19 cognitive changes.

### Inclusion criteria

(i) Adults diagnosed with post-COVID-19 syndrome, as defined by UK National Institute for Health and Care Excellence (NICE) ^1^;

(ii) First symptoms of COVID-19 from January to June 2021, reported by patients at admission;

(iii) Functional limitation according to the *Post-COVID-19 Functional Status* (PCFS) scale (above grade 0) at admission to the rehabilitation center;

(iv) Authorized participation in the study by an informed consent form;

(v) A neurological diagnosis other than post-COVID-19 syndrome.

### Exclusion criteria

(i) Physical or cognitive repercussions from other diagnoses prior to COVID-19;

(ii) Withdrawn authorization for participation;

(iii) Discontinued treatment or follow-up.

### Study design

This is a prospective study of cases for 12 months or more after acute COVID-19 infection.

Patients were admitted within 30 days after registering for evaluation and rehabilitation of the neurological consequences of COVID-19. Registration at the rehabilitation center was spontaneous and accessible to anyone via the institutional website without the need for a medical referral.

Data on post-COVID-19 symptoms, length of initial hospital stay, and comorbidities were obtained at medical admission by a structured assessment based on external medical reports. The admission protocol had a list of the most frequent symptoms of post-COVID-19 syndrome and, at the end, an open field with “other symptoms”. Personal and sociodemographic data, such as age, sex, education, and employment status prior to COVID-19, were also collected at medical admission.

After patients were admitted, evaluated by the team, and administered examinations, patients were referred to a rehabilitation program consisting of weekly 3-hour consultations over six weeks if a diagnosis of post-COVID-19 syndrome was confirmed. The program contained a series of exercises for strength, physical conditioning, and balance gain, groups for emotional support, coping, and cognitive stimulation, and guidance on post-COVID-19 syndrome, health, and quality of life if necessary. It also had an interdisciplinary team consisting of a physician, a nurse, a physical therapist, a physical education teacher, and a psychologist. During this follow-up, patients also received individualized care with the interdisciplinary team if necessary. An illustrative material with guidance on exercises and activities was given to all patients to encourage them to follow it at home.

Then, follow-up was organized according to individual demands. Patients who still showed symptoms continued treatment by specific individual or group consultations in person or online. Patients who progressed with the complete resolution of symptoms (or still showed very mild symptoms) were encouraged to reenter the community, resume their personal and professional lives, and incorporate the guidelines for a healthy lifestyle and regular physical activity that were developed during the rehabilitation program.

Patients were contacted by phone 12 months after developing the first symptoms of COVID-19.

Information of interest referred to:

(i) Medical admission: persistent post-COVID-19 symptoms (evaluated by a structured assessment).

(ii) Admission to the interdisciplinary rehabilitation program: (a) global functional status - PCFS; (b) cognitive assessment - *Montreal Cognitive Assessment*(MoCA); (c) emotional status - *Hospital Anxiety and Depression Scale* (HAD); (d) motor functionality - 5 times sit-to-stand test (TLS5x), Timed up and go test (TUG), 6-minute walk test (6MWT), and handgrip test; (e) perception of fatigue - *Fatigue Severity Scale* (FSS); (f) quality of life - *Short-Form Health Survey* (SF-36); and (g) indirect costs and loss of productivity by a specific questionnaire.

(iii) Phone contact 12 months or more after the first symptoms of COVID-19: (a) persistent post-COVID-19 symptoms by a structured assessment and (b) indirect costs and productivity loss by a specific questionnaire.

PCFS quickly classifies the global functional status of persons affected by COVID-19. It assesses participation in daily tasks and activities at home or at work/school and lifestyle changes. It has six gradations: PCFS0 (no symptoms), PCFS1 (negligible functional limitations), PCFS2 (slight functional limitations), PCFS3 (moderate functional limitations), PCFS4 (severe functional limitations), and PCFS5 (death) ^10^ ^,^ ^11^.

MoCA is a brief screening instrument that evaluates some cognitive functions, such as executive, visuospatial skills, and naming functions; memory retrieval, digits, sentence, and abstract reasoning and orientation, with a maximum score of 30 points (considering scores above 26 as normal) ^12^ ^,^ ^13^.

HAD consists of 14 questions, seven of which assess anxiety and seven, depression. Each item is scored on a scale from 0 to 3, with a total score of 21 points for each subscale (anxiety or depression). Scores below eight in each subscale indicate no anxiety or depression and those above nine, anxiety or depression ^14^ ^,^ ^15^.

TLS5x measures the time to get up from a chair as quickly as possible five times ^16^. TUG ^17^ consists of individuals getting up from a chair without the help of their arms and walking at the fastest and safest pace possible for three meters, turning around, returning, and sitting down again. In the 6MWT, the person walks along a flat corridor spanning a minimum length of 30 meters. The total distance traveled is measured at the end of the test ^18^. The handgrip test uses a dynamometer to measure grip strength in kilograms of force (Kgf) following Fernandes et al. ^19^.

FSS is a 9-item instrument that assesses fatigue severity in daily activities. Each item is scored from 1 to 7, with a score of one indicating strong disagreement and seven, strong agreement, with a possible total score from nine to 63 points. Fatigue is worse the higher the final score ^20^.

SF-36 consists of a 36-item multidimensional questionnaire with eight domains: functional capacity, physical aspects, pain, general health status, vitality, social aspects, emotional aspects, and mental health. Each has a score from 0 to 100 in which the higher the score, the better the person’s perception of their quality of life in that domain ^21^.

To obtain the indirect costs, a questionnaire was developed to find work status before COVID-19, during follow-up, the moment the person returned to work, and reasons for failing to resume work. The human capital methodology was used at the Brazilian Health Technology Assessment Network ^22^ recommendation to evaluate the indirect costs associated with productivity loss by estimating the number of working hours or days lost due to the disease multiplied by the Brazilian per capita income. indirect costs was also shown by multiplying the workdays lost due to the disease with the average income reported by patients during interviews.

### Data analysis

Data were descriptively and exploratorily analyzed by statistics (such as means and standard deviations) and percentages. The chi-squared and Mann-Whitney tests were used on SPSS, version 21 (https://www.ibm.com/), to evaluate associations between variables with a 5% significance level.

### Ethical aspects

This study was approved by the Research Ethics Committee of the SARAH Network (CAAE 50357921.3.0000.0022).

## Results

**Figure 1:**
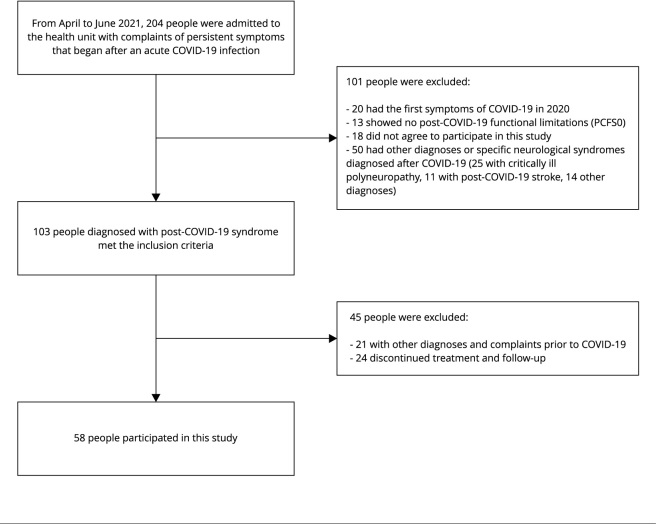
Flowchart of patient selection in this study.

The Fortaleza Unit admitted 204 people with complaints related to COVID-19 complications from April to June 2021. Of these, this study included 122 and excluded 24 people. Figure 1 shows a detailed flowchart of the patient selection process.

The final sample consisted of 58 participants with a mean age of 52.8±10.5 years, of which 62% were women (Table 1).

**Table 1:** Personal data, sociodemographic profile, employment prior to COVID-19, main comorbidities, and functional classification by the Post-COVID-19 Functional Status (PCFS) scale.

Characteristics	n = 58	%
Age (years)		
18-39	7	12
40-59	34	59
+60	17	29
Sex		
Female	36	62
Male	22	38
Schooling		
Higher education	19	33
Secondary education	24	41
Primary education	14	24
Illiterate	1	2
Pre-COVID-19 employment		
Formal market	13	22
Informal market with contributions to social security	11	19
Informal market with contributions to social security	18	31
Public server	4	7
Retiree/Pensioner	7	12
Unemployed	2	3
Domestic services	2	3
Student	1	2
Main comorbidities		
Arterial hypertension	25	43
Overweight/Obesity	16	28
Prediabetes/Diabetes	16	28
Previous psychiatric disorder	7	12
PCFS		
PCFS1	8	14
PCFS2	29	50
PCFS3	18	32
PCFS4	3	4

Note: information obtained at medical admission.

Most patients (67%) were admitted to the hospital for acute treatment of COVID-19 symptoms, with a 31-day mean length of stay (minimum 4; maximum 124 days). Of these, 60% required intensive care unit (ICU) admission for a 28-day mean length of stay (minimum 7; maximum 111 days) and 54%, intubation and invasive mechanical ventilation.

Table 1 shows personal data, sociodemographic profile, work status prior to COVID-19, main comorbidities, and functional classification according to the PCFS scale.

### Main persistent symptoms of post-COVID-19 syndrome

Patients were admitted an average of 132±72 days after showing the first symptoms of COVID-19. Patients suffered from chronic fatigue most often (64%), followed by arthralgia (51%), dyspnea (47%), lowered mood (44%), anxiety (44%), sleep disorders (44%), difficulty walking (37%), and memory alterations (36%).

**Figure 2:**
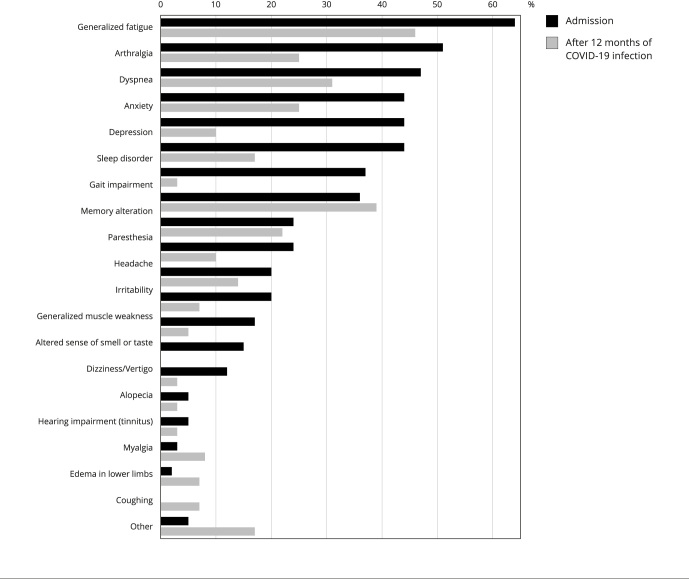
Persistent symptoms reported by patients on admission and 12 months after the first symptoms of COVID-19 (phone follow-up).

Telephone contact 12 months took place 451±31 days after the first symptoms of COVID-19. The most prevalent symptom referred to generalized fatigue (46%), memory impairment (39%), and dyspnea (31%). Figure 2 shows the most prevalent symptoms at admission and 12 post-COVID-19 months and Table 2, the main neurological symptoms grouped into cognitive and behavioral, motor, sensitive and painful, sensory (sight, hearing, smell, or taste), and sleep categories.

**Table 2:** Main neurological persistent symptoms reported by patients on admission and 12 months after the first symptoms of COVID-19 (phone follow-up).

Symptoms	Admission (%)	After 12 months of COVID-19 infection (%)
Cognitive and emotional	34	37
Anxiety	44	25
Depression	44	10
Memory alteration	36	39
Irritability	20	14
Motor	29	24
Generalized fatigue	64	46
Gait impairment	37	3
Generalized muscle weakness	20	7
Sensitive and pain	22	28
Arthralgia	51	25
Paresthesia	24	22
Headache	24	10
Myalgia	3	8
Sensory	13	5
Altered sense of smell or taste	17	5
Visual alteration	15	0
Dizziness/Vertigo	12	3
Hearing impairment (tinnitus)	5	3
Sleep	10	7
Sleep disorder	44	17

### Functional assessment and quality of life scales

The FSS assessment of fatigue obtained a 44.5±14 mean score, classifying 71.9% of patients as having moderate or severe fatigue. Regarding anxiety and depression, the HAD scale found probable or possible anxiety in 56% of patients and probable or possible depression in 46%. In cognitive screening, the MoCA scale showed a 22.0±0.7 mean score.

Regarding motor parameters, mean handgrip strength totaled 18.6±9.5Kgf on the right hand and 17.6±9.8Kgf on the left hand. The TLS5x test found a 11.2±3.8-second mean time and the 6MWT, a 352.4±154.2-meter mean distance.

Results for SF-36 quality of life dimensions showed a 59.8±20.1 general health status; 55.1±12.7 mental health, 51.3±22.5 functional capacity, 48.9±24.0 social aspect, 41.5±18.1 emotional aspect, 39.8±17.1 vitality, 26.9±39.1 pain, and 18.4±32.9 physical aspects mean scores.

### Indirect costs and return to work

In total, 79% of participants were working before contracting COVID-19, 31% were self-employed without contributing to the Brazilian Social Security Institute (INSS, acronym in Portuguese), 22% had formal contracts, 19% were self-employed and contributed to the INSS, and 7% worked as public servants (Table 1).

This study found 11,653 days of absenteeism, amounting to 8.3 post-infection months for professional reintegration.

Based on the ratio of BRL 1 = USD 5.50, the average per capita income in Brazil (BRL 1,367.00 in 2021), and the human capital method, the 58 participants totaled an indirect costs equal to USD 120,822.35 in the first post-infection year.

Estimates considering those who were economically active before contracting COVID-19 and participants’ average income (BRL 3,225.82) found an indirect costs equal to USD 227,821.00 (or BRL 1,253,016.02) in the first post-infection year, i.e., USD 4,847.25 (BRL 26,659.92) per person.

After 12 months of COVID-19 infection, 32% of people were unable to return to work and 95% reported persistent post-COVID-19 symptoms as the main reason for it.

People who returned to work within 12 months after contracting a COVID-19 infection performed better on the TLS5x and had better quality of life according to the SF-36 functional capacity, physical functioning, vitality, and pain dimensions, showing a statistically significant difference in relation to patients who were unable to return to work (Table 3).

**Table 3:** Comparison of scale and functional test results between patients who returned and who failed to return to work.

Evaluation parameter	Reintegration into work within 12 months post-infection (mean±SD)			p-value
	Yes	No	
MoCA *	22.5±2.8	22.7±3.6	0.794
HAD (anxiety) *	8.2±4.8	9.4±2.9	0.271
HAD (depression) *	6.6±4.3	8.2±3.5	0.227
FSS *	42.0±15.1	49.9±8.4	0.101
TLS5x **	9.7±3.0	13.0±4.0	≤ 0.05
TUG **	6.6±1.7	7.7±2.0	0.083
6MWT ***	377.6±166.2	317.4±151.2	0.127
Right hand dynamometer ^#^	20.7±9.7	17.2±8.9	0.275
Left hand dynamometer ^#^	19.5±9.8	16.4±11.3	0.216
PCFS *	2.1±0.7	2.4±0.7	0.158
SF-36: general health status *	62.7±21.6	56.0±17.7	0.370
SF-36: functional capacity *	57.4±21.2	41.7±18.1	≤ 0.05
SF-36: physical aspects *	30.6±39.9	1.4±5.9	≤ 0.01
SF-36: emotional aspects *	43.0±19.7	36.4±17.7	0.304
SF-36: vitality *	45.8±17.3	31.1±13.9	≤ 0.01
SF-36: mental health *	56.0±13.8	50.3±10.1	0.140
SF-36: social aspects *	54.8±24.7	44.4±22.0	0.155
SF-36: pain *	39.8±42.1	5.6±17.1	≤ 0.01

6MWT: 6-minute walk test; FSS: *Fatigue Severity Scale*; HAD: *Hospital Anxiety and Depression Scale*; MoCA: *Montreal Cognitive Assessment*; SF-36: *Short-Form Health Survey*; TLS5x: 5 times sit-to-stand test; TUG: timed up and go test.

* Mean scores;

** Time, in seconds;

*** Distance, in meters;

^#^ In Kgf.

## Discussion

Our study found persistent symptoms similar to those in the literature ^3^ ^,^ ^4^ ^,^ ^5^ ^,^ ^6^ ^,^ ^7^ but a higher frequency of these symptoms, which we believe stems from participants’ more disabling profile of such symptoms since they sought rehabilitation precisely to improve these symptoms and their impacts on their functionality, quality of life, and productivity. The 4-month post-COVID-19 followed found generalized fatigue (64%), arthralgia (51%), dyspnea (47%), anxiety (44%), depression (44%), sleep disorders (44%), gait disorders (37%), and memory alteration (34%) as the most reported symptoms.

An important contribution of this study refers to its finding that symptoms can persist and significantly impact persons’ life up to 15 months after the infection as 46% of participants reported generalized fatigue; 39%, memory alterations; 31%, dyspnea; 25%, anxiety; and 25%, arthralgia.

Huang et al. ^3^ reassessed 1,733 people who were discharged for COVID-19 six months after symptom onset, finding fatigue or muscle weakness (63%) and difficulty sleeping (26%) as the most common symptoms and that 23% of patients reported anxiety or depression.

A 2021 systematic review analyzed 33 studies with 8,293 people with persistent post-COVID-19 symptoms and found a 62% post-COVID-19 syndrome; 44%, fatigue, 40%, dyspnea; 34%, myalgia; and 33%, sleep disorder prevalence. Other symptoms included cough (22%), alopecia (20%), palpitations (20%), and arthralgia (13%) ^23^.

Malik et al.’s ^4^ systematic review analyzed 12 studies with 4,828 patients with post-COVID-19 syndrome and found fatigue (64%), sleep disorders (47%), dyspnea (39.5%), arthralgia (24.3%), headache (21%), anosmia (20%), and mental health (14.5%) as the main persistent symptoms.

Considering the presence of symptoms at various times after COVID-19, Augustin et al. ^24^ prospectively followed 442 and 353 patients over four and seven months after symptom onset, respectively, finding that 8.6% of patients had dyspnea; 12.4%, anosmia; 11.1%, ageusia; and 9.7%, fatigue four months after infection. After a 7-month median follow-up, symptoms remained similarly prevalent: 14.7% of anosmia, 13.6% of dyspnea, 14.7% of fatigue, and 11% of ageusia.

Of all the persistent symptoms in this study, only the prevalence of memory alterations increased over time; 36% of patients had it in the fourth month and 39%, in the 15th post-infection month. This study avoided explaining this increase but hypothesizes that it stems from the permanence of the deleterious effects of the virus in the brain areas related to memory or a perception change in patients as they were exposed to more complex tasks after resuming life activities. Braga et al. ^25^ developed a pioneering study on people who sought rehabilitation services due to memory, attention, and cognitive problem solving difficulties up to eight months after infection, finding that patients’ performance was below the reference values in all subscales and general scores of the *Barrow Neurological Institute Screen for Higher Cerebral Functions* (BNIS), especially in subtests for affect, memory, phonemic verbal fluency, and clock drawing, configuring the first to find difficulties with affect expression and perception in people with post-COVID-19 syndrome.

Some post-COVID-19 symptoms commonly reported in the literature were infrequent in this study, such as coughing ^23^, as no patient reported it in the first evaluation.

This study grouped patients’ main neurological symptoms, showing the repercussions of the post-COVID-19 syndrome on neurological systems and their impact by assessment scales, especially on cognitive-emotional, motor, and sensory-pain factors.

Based on scale results, 56% of patients had probable or possible anxiety and 46%, probable or possible depression, showing a 22 MoCA mean score. Regarding motor dimensions, 71.9% of patients had moderate or severe fatigue and altered handgrip, TSL5x, TUG, and 6MWT parameters.

Studies have also shown the functional changes due to post-COVID-19 syndrome by structured assessments: cognition and neuropsychology ^25^ ^,^ ^26^, anxiety and depression ^27^ ^,^ ^28^, strength and physical conditioning ^29^ ^,^ ^30^, and fatigue ^31^ ^,^ ^32^. Our study also correlated functional scale results with work reintegration after COVID-19, finding that patients who resumed working had better TLS5x results.

The repercussions on quality of life were very significant, with physical aspects (18.4), pain (26.9), and vitality (39.8) configuring the main compromised dimensions. Since each dimension has a maximum score of 100 (with the highest score corresponding to a better perception of quality of life), these results evince the negative impact of COVID-19 on people’s health and lives.

Physical disability and reduced health-related quality of life are common repercussions after COVID-19 infection ^7^ ^,^ ^33^. Malik et al. ^4^ found that patients reported a low perceived quality of life, scoring 59% on the EQ-VAS scale. In the EQ-5D-5L questionnaire dimensions, 36% of participants perceived a low quality of life in mobility; 8%, in personal care; 28%, in usual quality; 42%, in pain/discomfort; and 38%, in anxiety/depression. Meta-regression analysis showed that worse quality of life was significantly higher among patients admitted to ICU and with symptoms of fatigue. Huang et al. ^3^ found that patients generally classify quality of life with an 80% final score, with 27% of people reporting pain and discomfort; 23%, anxiety and depression; and 7%, mobility problems.

Cao et al. ^34^ followed 81 patients for three months after hospital discharge. Participants generally had a mild COVID-19 profile (only 13% required ICU and only one, intubation), unlike the profile in this study. The quality of life in the SF-36 was significantly impaired in the physical functioning and social functioning domains when compared to normal individuals of the same age. Differences between age groups showed impaired emotional state in the 41-64 age group; pain and mental health in the 41-64 age group; and emotional state in the 18-64 age group.

In this study, 32% of people who worked before contracting COVID-19 were unable to return to work even 15 months after the infection, and patients who reintegrated professionally required more than eight months to return. Cases without reintegration into the workplace showed a worse perception of quality of life regarding functional capacity, physical aspects, vitality, and pain. Regarding these results, we raise the possibility that the physical limitations of post-COVID-19 syndrome (such as fatigue, pain, muscle weakness, and poor physical conditioning) negatively impact participation and thus quality of life and job reinsertion.

Thus, indirect costs greatly exceeded the Brazilian socioeconomic reality, totaling hundreds of thousands of dollars for the 58 followed patients: USD 227,821.00 in the first year after infection, corresponding to USD 4,847.25 per person according to the reported income methodology. As this study only considered the indirect costs of work absenteeism, these costs would certainly be much higher if it had considered reduced work productivity, relatives leaving work to provide care, and premature deaths due to COVID-19.

Few studies report on the indirect costs of COVID-19 considering patients’ perspective. Ghaffari-Darab et al. ^35^ analyzed the costs of 477 individuals admitted to a hospital in Iran and found a 21-day absenteeism average, estimating an average indirect cost of USD 11,634 per person by including loss of income from premature death, lower productivity due to hospitalization, and absenteeism during recovery.

A study with 19,086 U.S. military staff found that 299 (2%) required at least one hospitalization for COVID-19 (which averaged 4.8 days) ^36^. Post-hospitalization recovery lasted an average of 11 days after hospital discharge, with a USD 4,782,790 total indirect costs and an average of USD 3,576 per person from recovery to return to work.

Maltezou et al. ^37^ studied 3,332 healthcare providers and estimated EUR 1,735,830 total costs, with absenteeism representing a large part of this total (80.4% of all expenses, equivalent to EUR 1,388,664).

### Study limitations

Participants sought rehabilitation as more severe post-COVID-19 syndrome persistent neurological symptoms impacted their lives. Thus, our results fail to reproduce the general status of post-COVID-19 syndrome due to the selection bias in the studied sample.

However, results probably denote cases with the more severe neurological symptoms of the disease.

This study also ignored the relation between the initial severity of COVID-19 and persistent symptoms. Although patients had more severe cases of COVID-19 at the beginning of the infection (64% were hospitalized for an average of one month; 60% were in the ICU for an average of 28 days, and 54% were intubated), developing this association is impossible as our research design is inappropriate for this.

Another limitation refers to the lack of evaluation parameters for sensitivity and pain, which were significant changes at follow-up. The SF-36 scale pain dimension partially compensated this limitation, which, according to patients’ perception, attested to pain and its impact on quality of life and return to work.

The study unfortunately failed to apply its functional scales 12 months after the infection in all patients, data that would be interesting to compare to initial evaluations.

## Conclusion

The most frequent persistent symptoms in people with post-COVID-19 syndrome who sought rehabilitation at the Fortaleza unit of the SARAH network refer to generalized fatigue, arthralgia, dyspnea, anxiety, depression, and sleep disorders, impacting patients’ cognitive, emotional, motor, and quality of life function. These symptoms persisted for more than 12 post-infection months, with a greater frequency of generalized fatigue, memory impairment, dyspnea, anxiety, and arthralgia. Participants reported memory alterations more often 12 months after the infection than in their initial evaluation.

Patients with the more disabling symptoms of post-COVID-19 syndrome showed significant difficulties returning to work, demanding, on average, more than eight months for professional reintegration and totaling USD 4,847.25 indirect costs per person in one year.

Better results in the TLS5x and better perceived quality of life in functional capacity, physical aspects, vitality, and pain in the fourth post-infection month were related to return to work. Some cases showed no return to work even 15 months after the infection.

These results show the long-term repercussions of post-COVID-19 syndrome on cognitive, emotional, and motor functions, evincing its significant negative impact on affected people’s functionality, health, labor reintegration, and quality of life. Rehabilitation treatment by interdisciplinary health teams is essential due to the several compromised neurological dimensions as is long-term treatment for some cases as symptoms may last for more than 12 months post-infection.
